# Transcription Factor Evolution Associated with Social Transitions in Blattodea

**DOI:** 10.1007/s00239-026-10328-1

**Published:** 2026-07-23

**Authors:** Alun R. C. Jones, Elias Dohmen, Juliette Berger, Frédéric Legendre, Mark Harrison, Erich Bornberg-Bauer

**Affiliations:** 1 Institute for Evolution and Biodiversity, University of Münster, Münster, Germany; 2 Newcastle University, Population Health Sciences Institute, Faculty of Medical Sciences, Newcastle upon Tyne, UK; 3 Institut de Systématique, Évolution, Biodiversité (ISYEB), UMR 7205, Muséum national d’Histoire naturelle, CNRS, Sorbonne Université, EPHE-PSL, Université des Antilles, 57 rue Cuvier, F-75005 Paris, France; 4 Research Center for Health and Life Sciences, Coventry University, Coventry, UK; 5 Department of Protein Evolution, Max Planck Institute for Biology Tübingen, Max-Planck-Ring 5, Tübingen, Germany; 6 Theoretical Sciences Visiting Program, Okinawa Institute of Science and Technology Graduate University, Onna, 904-0495 Japan

**Keywords:** Evolution, Eusociality, Transcription factors, Blattodea, Regulation

## Abstract

The phenotypic diversity between castes in social species has been associated with increased regulation of gene expression. while the regulatory role of transcription factors (TFs) in the social transitions of Hymenoptera (bees, ants and wasps) is well studied, it remains poorly explored in Blattodea (cockroaches and termites). By analysing three cockroach and five termite genomes, we show that while Blattodea share some convergent regulatory signatures with Hymenoptera, their transition is mostly characterised by distinct regulatory changes. We identified convergent patterns such as TF families with more relaxed selection compared to intensified selection and lineage-specific gene family expansions in termites, which has also been reported in Hymenoptera. There are also key differences in Blattodea TF regulation in comparison with Hymenoptera with contractions in TF gene families and no compensatory changes in TF DNA binding motifs either in frequency or diversity in TF promoter regions. Furthermore, we show that an increase in social complexity associates with greater diversity in TF activating domains, one of the evolutionary and structural building blocks of TFs; meanwhile, DNA-binding domains, undergo very little change. This study highlights similarities in social transitions between Hymenoptera and Blattodea, with evidence of large changes in transcriptional regulation followed by lineage specific adaptations. Our results indicate that at least in the case of Blattodea, the transcriptional diversity linked to social complexity is not attributable to transcription factors alone, but is instead likely driven in combination with alternative mechanisms.

**Supplementary Information:**

The online version contains supplementary material available at 10.1007/s00239-026-10328-1.

## Introduction

In their book Major Transitions in Evolution, Smith and Szathmáry (2010) highlighted that eusociality is one of the major transitions in evolution where individuals forgo their own ability to reproduce to cooperate as a group to form a more complex being, analogous to genes integrating to form genomes. The defining characteristics–cooperative brood care, reproductive division of labor, and overlapping generations–have been observed in a wide variety of taxa, including mammals, shrimps, and insects, such as bees, ants, wasps and beetles (Crozier and Pamilo 1996; Revely et al. 2024; Legendre and Grandcolas 2018; Jennifer et al. 1994; Solomon et al. 2021). Blattodea (cockroaches and termites) and other groups exhibit a spectrum of sociality, including subsociality which involves parental care and gregarious behaviour (Hölldobler and Wilson 2009; Crozier and Pamilo 1996; Legendre and Fabien L Condamine 2018). While Hymenoptera (bees, ants and wasps) have historically dominated eusocial research (Sandra et al. 2015), possibly due to historical and geographic research bias as well as having multiple origins of eusociality (Peters et al. 2017). Blattodea offers a unique comparative model where eusociality arose only once in the termites. Blattodea spans a full continuum of social complexity, from solitary and gregarious to subsocial, primitively eusocial and highly eusocial lifestyle. (Roth and Nalepa 2009; Legendre and Grandcolas 2018; Bornberg et al. 2018; Fouks et al. 2023).

Blattodea possesses distinct biological features that contrast sharply with Hymenoptera. Unlike the holometabolous Hymenoptera, termites are hemimetabolous. Furthermore, termite colonies also consist of both diploid males and females, including long lived reproductives of both sexes (Tasaki et al. 2021) with XY sex chromosomes (Lacy 1980) while hymenopteran colonies are typically founded on a haplodiploid system with female workers. These fundamental differences make Blattodea an essential group for disentagling universal molecular signatures of social evolution from those that are clade-specific.

Exploring the molecular details underlying the evolution of Blattodea has only become feasible recently, with five termite and three cockroach genomes having been published to date of this analysis (Mark et al. 2018; Bornberg et al. 2018; Terrapon et al. 2014; Fouks et al. 2023; Meng et al. 2016; Li et al. 2018; Shigenobu et al. 2022), which we consider as key species for this study. All of these have shown that the genome size of termites are smaller then cockroaches both in size and gene content, in keeping with the trends found in hymenoptera social evolution. In Blattodea, other remarkable insights have identified mechanisms of viviparity (live bearing) and genomic changes related to social evolution, including the regulation of chemical perception, a hallmark of insect societies used to recognise nestmates, caste status or sense food sources. Interestingly, termites exhibit significant expansions in ionotropic receptors (Mark et al. 2018), in contrast to the expansion of odorant receptors found in Hymenoptera (Zhou et al. 2015), although the later may be rather flight loss in in hymenopteran females (Gautam et al. 2024). The divergent expansion of two chemoreceptor families indicates that while social transitions required conserved functional shift in chemical perception the specific underlying molecular mechanisms driving those shifts vary between orders. Another convergent pattern includes alterations in methylation patterns associated with caste-specific gene regulation (Mark et al. 2022; Lyko et al. 2010; Glastad et al. 2016), although this relationship is not necessarily consistent (Glastad et al. 2017). Specific regulatory pathways, such as the juvenile hormone pathway, involved in caste determination, also convergently changed in both Blattodea and Hymenoptera (Mark et al. 2018; Robinson and Vargo 1997). However, a more general understanding of regulatory changes related to social evolution in Blattodea has not been conducted. It remains unclear whether similar convergent patterns exist in termites. To this end, Harrison et al. (2018) identified expansions in zinc fingers in termites (Mark et al. 2018) but changes in regulatory regions were not studied.

To bridge this gap, the present study focuses specifically on transcription factors (TFs), whose DNA binding domain and secondary activating domains control gene expression (Latchman 1997). Within Hymenoptera TF influence in social evolution has revealed convergent patterns of enrichment of TF binding sites in regulatory DNA regions, accompanied by significant gains in particular gene families (Simola et al. 2013; Kapheim et al. 2015).

This study aims to identify evolutionary changes in transcription factors and their regulatory potential that can be associated with social evolution in Blattodea. Utilizing eight published genomes, we investigate alterations in TF-mediated regulation. Specifically, using protein family (PFAM) information, we examine changes in TF number, binding structure, binding targets, and the interplay between all of those. Our goal is to elucidate key patterns for complex social transitions in Blattodea. By comparing these findings to analogous patterns in Hymenoptera, we aim to create a more comprehensive understanding of the genetic regulatory changes associated with social evolution.

## Materials and Methods

### Transcription Factor Identification

The defining features of TFs are the DNA binding domains and a secondary activating domain (Latchman 1997). These protein domains can be identified from known modular DNA sequences allowing the identification of conserved protein functions and evolution (Sonnhammer et al. 1997; Apic et al. 2001). For this study, transcription factors were identified using 70 Unique DNA binding PFAM domains (Mistry et al. 2021) found in *D. melanogaster* transcription factors from FlyBase. The majority of TF classes arose before the emergence of multicellularity (Mendoza et al. 2013; Bernard M Degnan et al. 2009). Therefore *D. melanogaster* share the vast majority if not all of the TF classes with Blattodea. We chose three cockroaches: *Blattella germanica *(84.1% BUSCO completeness), *Diploptera punctata *(97.6% BUSCO completeness) and *Periplaneta americana *(97.6% BUSCO completeness) alongside five termite species: *Zootermopsis nevadensis *(98.4% completeness), *Cryptotermes secundus *(93% BUSCO completeness), *Reticulitermes speratus *(98.5% BUSCO completeness), *Coptotermes formosanus *(99.83% BUSCO completeness) and *Macrotermes natalensis *(97% BUSCO completness). Additionally two outgroup species were used; *Frankliniella occidentalis *(99% BUSCO completeness) and *Eriosoma lanigerum *(97% BUSCO completeness) (Li et al. 2018; Biello et al. 2021; Fouks et al. 2023; Mark et al. 2018; Itakura et al. 2020; Poulsen et al. 2014; Rotenberg et al. 2020; Shigenobu et al. 2022; Terrapon et al. 2014). These species were chosen not only because at the time of the study they were all the genomic resources available but they also represented a large spectrum of sociality with solitary, gregarious, primitively and highly eusocial species being used. The outgroups *E. lanigerum *, the woolly apple aphid and *F. occidentalis *the western flower thrips were selected because of the quality of their genomes, their phylogenetic position to Blattodea and to keep consistency with previous studies. To annotate their domains, pfam scans were run using the longest isoform protein sequence, and genes with any of the 70 DNA binding domains were identified as potential transcription factors, which resulted in 12,630 potential TFs. Using those potential TFs, orthogroups were identified using Orthofinder2 (David et al. 2018)(including *D. melanogaster*) in a total of 1621 potential TF containing orthogroups). Orthogroups containing genes identified as potential TFs were used for all analyses.

### Multiple Sequence Alignment

Amino acid multiple sequence alignments of TF containing orthologs were run using GUIDANCE(2.02) (Sela et al. 2015) and MAFFT(v7.397) (Katoh et al. 2009) using a global pair alignment with max iterations of 1000. Unfiltered protein alignments were transformed into nucleic acid sequences using Pal2Nal (Suyama et al. 2006) and aligned but only using 200 iterations and the resulting alignments were filtered according to the amino acid alignments using a script from Fouks et al 2021, which removed all alignments below a 90% confidence score from the final alignment (Fouks et al. 2021).

### Gene Family Dynamics and Selection

To identify TF family expansions and contractions, CAFE5 (Fábio et al. 2021) was used using the Orthofinder2 results. Only orthogroups where TFs had been identified in Blattodea species were used. These results were formatted using a custom bash script. A multi-lambda model was used with two lambda values estimated, one for the termite branches and one for the rest (Fábio et al. 2021). GO terms were annotated using the PFAM2GO (Mitchell et al. 2015) from the sequences used in CAFE and a GO enrichment was done using TopGO with the CAFE sequences as the background universe and a significance threshold of a p-value of <0.05 (Alexa and Rahnenfuhrer 2017).

All 45 single-copy orthologs that were identified were used to investigate signals of selection concerning social evolution. A species tree was constructed by concatenating all single copy alignments and then running IQtree2(2.1.3) (Bui Quang Minh et al. 2020). Branch-specific relaxed selection was investigated using RELAX (Wertheim et al. 2015), branch-specific selections using aBSREL (Smith et al. 2015) with the termites as the selected branch and sites of episodic diversifying selection using MEME (Murrell et al. 2012). Significance was determined using a pvalue of <0.05 for MEME and aBSREL and a false discovery rate <0.05 for RELAX. Visualisation of selection was done through Hyphy vision website.

### Protein Family Dynamics

PFAM domain rearrangement events were computed with DomRates (Dohmen et al. 2020) and visualised with the DomRates visualisation script. The total number of DNA binding domains was extracted using custom python and bash scripts and then clustered together using the R "pheatmap" package (Kolde 2015).

While genes may duplicate, it does not mean that the resulting duplications have the same PFAM domain arrangements (Dohmen et al. 2020). To understand the dynamics between gene duplication and how sociality might be linked to domain changes, the relation between the number of genes in an orthogroup and the number and type of PFAM domains was modeled using a Bayesian approach. An Ornstein–Uhlenbeck (OU) covariance matrix was constructed using the species tree and the ape package vcv function (Paradis and Schliep 2019). Two different full multilevel negative binomial phylogenetic models were constructed using orthogroup and species as mixed effects to account for the multiple species level observations. Both full models contained fixed effects for each species’s orthogroup. One model contained the total number of DNA binding domains and the total number of activating domains, while the other contained the number of unique DNA binding domains, and the number of unique activating domains. A modifier term with sociality was added for each of these fixed effects in addition to the fixed effects. Each species was given a value for the different sociality indices: solitary, gregarious, primitively eusocial and highly eusocial based upon established traits determining their levels of social organisation (Sumner et al. 2018; Legendre and Grandcolas 2018). This had: *M. natalensis *, *R. speratus *and *C. formosanus *as highly eusocial, *C. secundus *and *Z. nevadensis *as primitively eusocial, *B. germanica *, *P. americana *and *D. punctata *as gregarious and the outgroups as solitary. The model was run using brms(2.20.1) (Bürkner 2017) with four chains on four cores using weakly regularising priors and a warmup of 1500 iterations and sampling of 3000. Diagnostic plots were looked at with DHarma (Hartig 2016). Two full models were reduced and compared using leave-one-out-cross-validation (LOO) (Vehtari et al. 2017). Analysis was run in R(4.3.1) using the tidyverse, dplyr, ggplot and bayesplot packages (Wickham 2016; R Development Core Team 2010; Gabry et al. 2019; Wickham 2011).

### Promoter DNA Binding

Promoter regions were extracted by taking a region of 2000bp upstream of the gene’s start codon based upon the length of known promoters (Kristiansson et al. 2009) using GTF tools and Bedtools (Buffalo 2015; Li et al. 2022; Quinlan and Hall 2010). These were then split into general promoters and promoters for TFs. Using the JASPAR insect-specific TF binding motif database (Jaime et al. 2022), DNA binding motifs were first identified using XSTREME, which combines both MEME and STREME with default parameters (Bailey et al. 2015) and only designated as a match with q-values <0.05, otherwise there were deemed ’novel’. Motifs were checked for enrichment using SEA, which shuffles each sequence in the input set to create a control set and uses a Fisher exact test to test for enrichment against the control set. Enrichment was considered significant if it had a q-value of less than <0.05 (Bailey and Grant 2021). Motifs were compared using TOMTOM (Gupta et al. 2007). Motif comparison was done in R using JASPAR packages. The Shannon diversity index for each species was calculated using the vegan package (Oksanen et al. 2001). To find if the diversity index was different between cockroaches and termites, we ran a permutation test accounting for TF number. To account for the relationship between TF number and diversity index, we ran a linear model between gene number and diversity index using R’s lm function (R Development Core Team 2010). The observed difference in residuals between cockroaches was calculated. The labels of cockroach and termite were randomly shuffled 10,000 times and a distribution of differences between group residuals was calculated. The p-value, is calculated as the proportion of permuted differences at least as extreme as the observed difference.

## Results

### TF and Orthology Identification

#### Selection

To see if TFs in Blattodea follow similar patterns of selection concerning social evolution as those found in Hymenoptera, we looked at branch specific relaxed and episodic diversifying selection as well as site-specific episodic diversifying selection in 45 single copy ortholog gene alignments. RELAX (Wertheim et al. 2015) identified seven orthologs undergoing relaxed selection, all of which contain all Blattodea species, with no genes undergoing intensified selection. We also looked at the function of these genes in *Drosophila* as it is the most studied insect in terms of gene function, even though *Drosophila* is holometabolous while Blattodea are hemimetabolous.

Of the seven relaxed genes, four had variations in DNA binding domains. The zinc finger *Klumpfuss*, which is involved in neuroblast identification (Kaspar et al. 2008), includes a change in the C2H2-type zinc finger domain in *M. natalensis *. * Cytochrome c oxidase assembly factor 10* (*Cox10*), which is involved in heme biosynthesis and respiration (Miozzo et al. 2022), gains a homeodomain in *M. natalensis *, but does not have a DNA binding domain in other species. There is a gain of C2H2 zinc fingers in the vacuolar protein sorting protein 16B (*Vps16B*) in *B. germanica * protein but this is absent in other species. This gene is important for maintaining vacuole stability and structure (Akbar et al. 2011). Three of these seven genes; *snoo*, *tango* and *klumpfuss*, have known neuro-developmental functions. *tango* is involved in regulating *breathless* which regulates trachea development and axonogenesis (Sonnenfeld et al. 1997). *snoo* binds to a different TF which changes its affinity resulting in antagonism of the product of *dpp* and facilitation of Activin signaling (Tsuda et al. 2002). *vrille* (*vri*), a bzip TF, also regulates *dpp* but as an enhancer. *dpp* is important for late stage wing development (Schertel et al. 2015; Ohde et al. 2022) (Supplementary 5).

aBSREL, which is an adaptive branch-site rel test (Smith et al. 2015), that identified one gene with positive selection in two termite branches with six other genes with selection in one termite branch. All branches identified for positive selection were terminal branches. The orthogroup with selection in two branches were in the *R. speratus *and *C. secundus *terminal branches is *Lim3*, a zinc finger and homeodomain transcription factor that regulates neuronal subtype, particularly motor neuron identity. Two genes, *Vps16* and *ATPSynthase*, only gained DNA binding domains in *B. germanica *and *P. americana *, so are not TFs in termites. *Macrotermes natalensis *has positive selection in *lmd* or *lame duck*, which is important for the specification of myoblasts (Duan et al. 2001), and in *abstrakt*, which is a DEAD box helicase, involved in processes such as axon asymmetric cell division (Irion et al. 2004). *Macrotermes natalensis *also was observed with selection in the Zinc finger *CG7987* which is associated with negative regulation of transcription (Tegeder et al. 2019), while *C. formosanus *was found to have positive selection in Helix-loop-helix transcription factor *tango*.

MEME was used to identify sites undergoing site specific episodic diversifying selection and identified 22 sites of episodic and pervasive selection (Murrell et al. 2012). Five sites appear to undergo episodic diversifying selection. Three of these sites are in *Vps16* and one in *CEP104*, which is important for centriole elongation. Both orthgroups only gained DNA binding domains in cockroaches. Episodic positive selection was identified in *UPf1*, which is involved in mediating nonsense mRNA degradation. Of the 11 orthogroups in which the 17 sites were identified with purifying selection, only 7 have DNA binding domains in Blattodea. One of them only gained a DNA binding domain in *M. natalensis *and another gained a DNA binding domain in *P. americana *. Orthogroups with DNA binding domains across Blattodea include: *tango*, *CG7897*, *CrebB*, *PSEA-binding protein*, *Inverted repeat protein* and *Lim3*. *PSEA-binding protein* is part of the small nuclear RNA activating complex which is involved in activating transcription of snRNA genes (Ko-Hsuan et al. 2009). *CrebB* is a transcription factor important for controlling genes important for long-term memory formation (Fropf et al. 2013). *Inverted repeat protein* forms part of the Ku complex and is involved in double strand break repair and telomere maintenance (Merigliano et al. 2017).

### Gene Family Changes

**Fig. 1:**
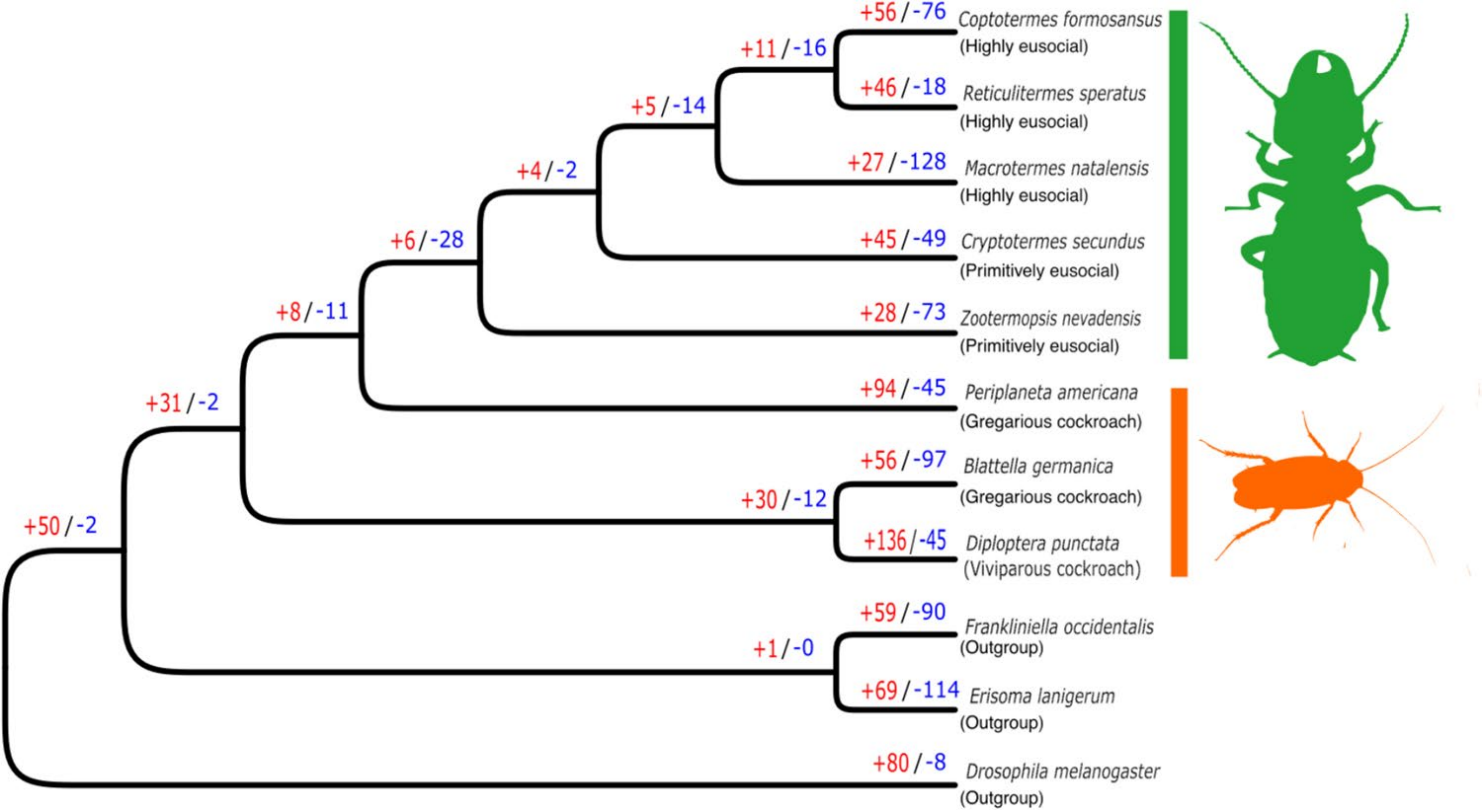
Cladogram of phylogenetic tree depicting all the species used, whether they are cockroaches or termites and their type of social organisation. Numbers in red are the number of TF containing orthogroups undergoing gene family expansions and the blue numbers are the TF containing orthogroups undergoing gene family contractions

At the origin of termites we found 28 gene family contractions and 6 gene family expansions (Fig. 1). We then looked at significant gene family changes, which CAFE uses a likelihood ratio test and a p-value of < 0.05 to determine. In termites, at any of the branches within the clade, there were 75 significant gene family contractions compared to 63 expansions. This shows that the majority of gene family changes occur after the social origin and contractions dominate at the phylogenetic origin of eusocial evolution.

Given the high number of contractions, we next looked into the prevalence of contractions within the termite lineages at the terminal branches. Four out of the five termite species have more contractions than expansions. Those gene families undergoing expansion are lineage-specific.

There are 21 orthogroups found to only undergo contractions, while only 9 orthogroups undergo expansion. Genes families that we see undergoing expansions at the termite node include *Neuroectoderm-expressed 2*, *Rfx*, *elbow b*, *noc* and gemini (Also in *Ubiquitin-63E* and*CG11700* but doesn’t have TF activity in fly). To understand if there were similar processes affected by gene family contractions we conducted a GO term enrichment analysis. The GO term enrichment of contracted orthogroups did not show any particular function or process that was enriched.

### PFAM Dynamics

**Fig. 2:**
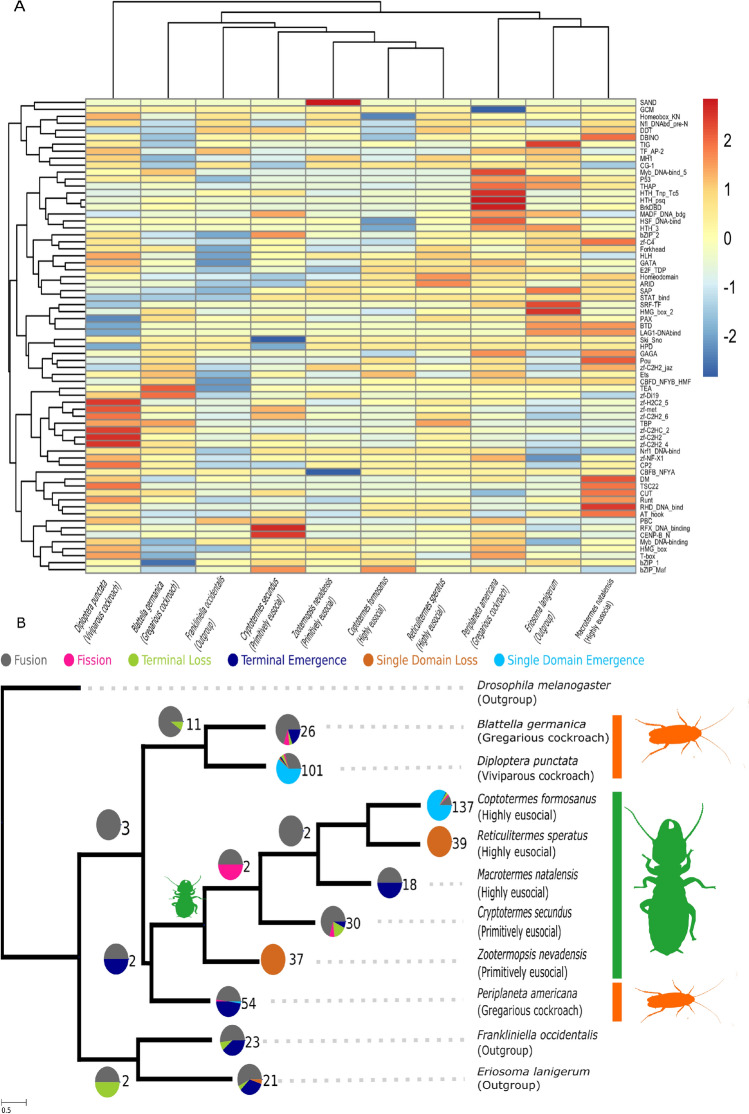
**A** Heat map showing the prevalence of DNA binding domains. The number of domains has been standardised across the species representing the number of DNA binding domains within a species relative to the total mean no of domains within the species.** B** Figure depicting the output from DomRates. The numbers represent the number of domain rearrangements while the pie charts show the proportion of event types. The origin of termites is marked by the smaller green termite silhouette

There were no obvious overall grouping in DNA binding domain numbers based upon the heat map, there were no clear increases or decreases in DNA-binding domains among TFs that could be observed in termites (Fig. 2A).

To see if there were key changes in function brought about via changes in domain modularity, we looked at protein domain rearrangements using DomRates (Dohmen et al. 2020). DomRates did not reveal any clear patterns in TFs domain rearrangements as the type of domain rearrangement is varied in termites. Also at the emergence of the termites, there were no domain changes (Fig. 2B).

**Fig. 3:**
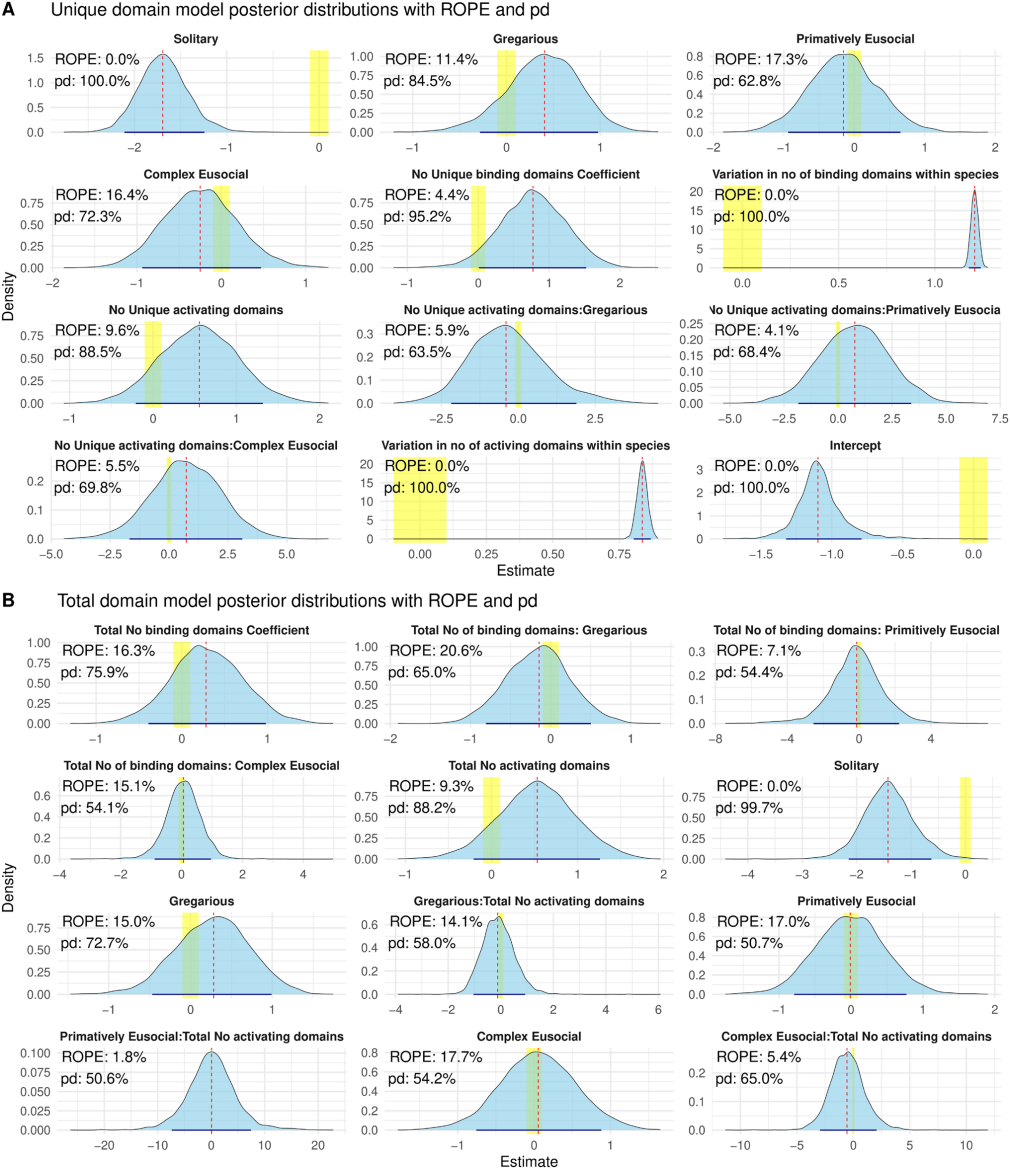
Here we are some of the results of the phylogenetic modelling looking at how total number of domains and the number of unique domain predicts the gene size of an orthogroup, which can be seen in Supplementry Table 4 and 4. We are showing the posterior distributions of a selected number of coefficients of the best fitting models, based upon LOO comparisons. The yellow region is the region of posterior equivalence (ROPE) between -0.05 and 0.05 with the percentage being the percentage of the posterior distribution that lies within this region. pd percentage indicates the probability of direction (PD). (**A**) Shows the best fitting model based upon the total unique number of domains within the gene family. (**B**) Shows the best-fitting model based upon the number of unique domains within the Gene family. There is a positive shift from interaction between the No Unique activating domains and Gregarious species and Eusocial species. Indicating there are more unique activating domains in Eusocial species orthogroups

The modeling analysis to identify the relationship between PFAM domains and orthogroup gene family size showed that the best-fitting model in the unique protein domains model set included a moderating effect with social index and activating domains. (Supplementary Table 1). The best-fitting model for the total domain model set was the full model set (Supplementary Table 2). The LOO model comparison shows that a moderating effect with social index is informative in predicting orthogroup size. Figure 3 shows some of the coefficients represented in the two tables and their posterior distribution. It shows the relationship between; the Pfam domain number and social index of the species with the number of genes within an orthogroup. We see a negative direction for the moderating effect with unique activating domains in solitary and gregarious species, while with primitively eusocial and highly eusocial species this moderating effect becomes positive (Fig. 3A). A similar pattern is seen in the total domains model with the social index moderating effect that shifts the coefficients in a positive direction for the activating and DNA binding domains (Fig. 3B). This indicates a small but meaningful positive relationship between the variety of domains and the total number in social species. Both sets of models show positive relationships between the number of domains and the orthogroup size meaning that several predictors have collinearity.

### DNA Binding Motifs

**Fig. 4:**
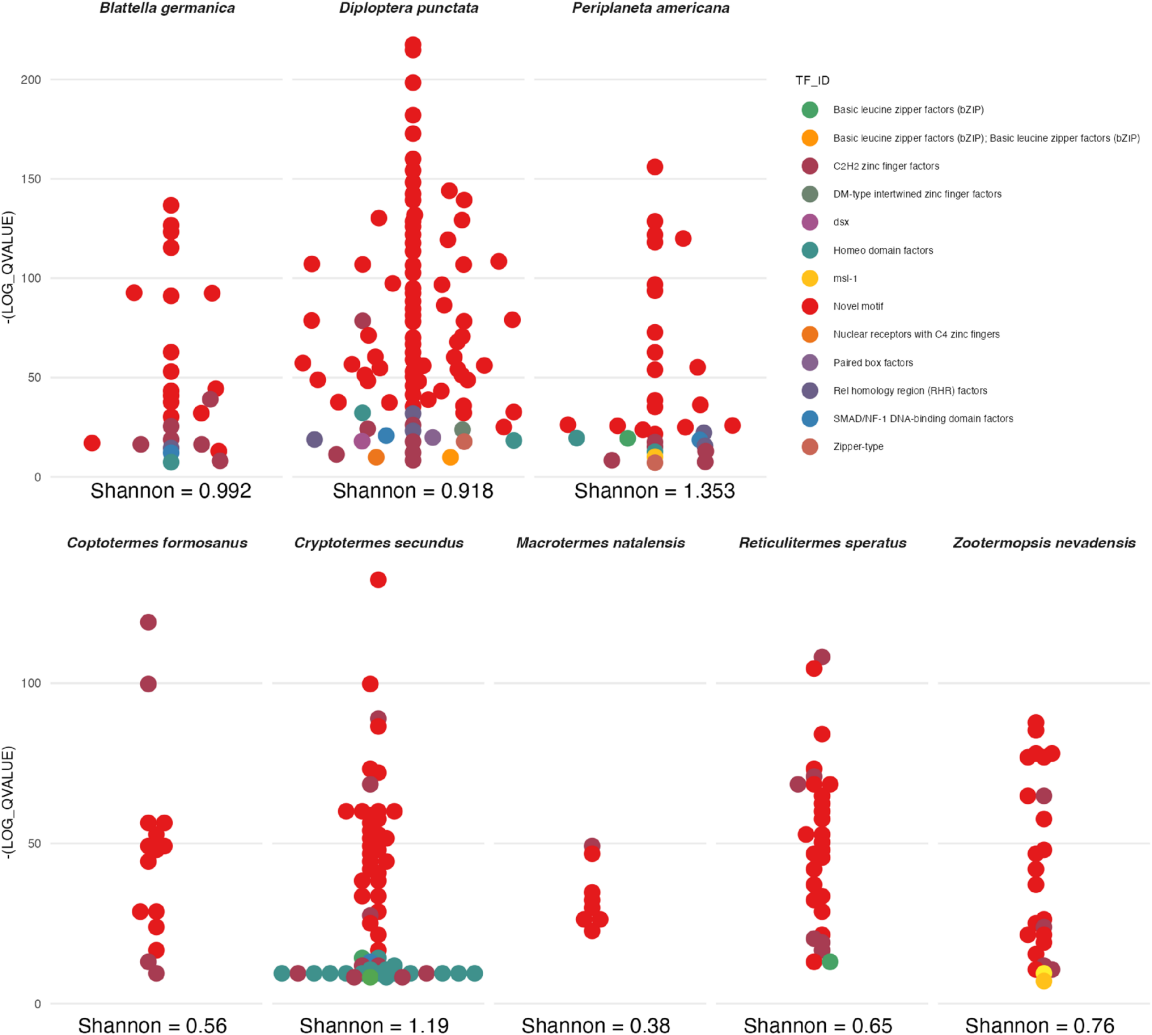
Enrichment of DNA binding domain motifs in 2kbp promoter regions of TFs in cockroaches as well as termites, identified using SEA from the XStreme package. The Shannon diversity index is displayed for each species. The y axis is the - log q value showing the significance of motif enrichment

The analysis on the enrichment of DNA binding motifs in promoter regions suggested cockroach TFs being enriched with more motifs and by more diverse motifs compared to termites (Fig. 4A). Given the difference in the number of TFs between the species, we wanted to see whether this was due to a product of differences between groups or to the reduction in the number of TFs (Fig. 1). When we compared the difference between cockroaches and termites the apparent difference was not significant in either TF promoters (p-values of 0.75) and in the other genes (p-value of 0.69).

## Discussion

One of the key findings of this study is that, unlike Hymenoptera, social termites show no significant change in promoter diversity or enrichment compared to cockroaches. While expansions in motif binding regulation correlat with higher levels of social organisation in Hymenoptera (Simola et al. 2013; Kapheim et al. 2015), our results suggest that this is not the case for Blattodea. Therefore, increasing diversity of regulation does not necessarily stem from increasing TF binding site availability. So this should be considered in the context of other TF-related changes, such as changes in TF gene family and domain composition. Our model indicates that diversity is instead created by changing domains, at least through the variation in activating domains (Fig. 3A). This suggests that (mechanistically) upstream regulation via signaling pathways has changed and diversified, an aspect future studies should explore.

While the difference between cockroach and termite may be attributed to overall differences in TF numbers, two considerations need to be made. First, the limited number of species in this study makes it difficult to rule out differences in promoters. Second, given what is known in Hymenoptera (Simola et al. 2013; Kapheim et al. 2015), we predicted that a reduction in TFs would be compensated for by an increase in TF promoter regulation. Contrastingly, we observe a reduction in TFs (Fig. 1) with no corresponding increase in TF regulation (Fig. 4). This reduction in TFs aligns with general trends in Blattodea, where termites have smaller genomes than cockroaches despite selective expansions in genes such as ionotropic receptors and immune related genes (Mark et al. 2018; Terrapon et al. 2014; Shigenobu et al. 2022; Alun et al. 2026; Liu et al. 2025). Our promoter results, coupled with the overall reduction in TF families at the origin of termites (Fig. 1), indicate that increased TF regulation may not be a necessity for transitions towards eusociality. Instead, a general large regulatory shift followed by lineage-specific changes appears more likely. This trend is also observed in Hymenoptera (Simola et al. 2013), where addedgeneral regulation changes, such as gene family expansions, occur alongside lineage-specific gene composition changes. Similarly, the lack of convergence in the orthogroups undergoing significant expansions in termites suggests a pattern of large changes in gene regulation followed by alterations in lineage-specific gene composition.

While there are no large-scale changes in domains at the origin of termites, terminal branches in termites show frequent changes in domains (Fig. 2A). Regarding transcription factor binding sites in TF promoter regions, although there is no significant difference between cockroaches and termites, *C. secundus *is a clear outlier (Fig. 4). Due to data availability, we limited this study to key previously studied species; future studies should use more comprehensive data sets to understand if the reduction of enriched sequences results from the loss of TFs with enriched promoters or a more general loss of binding sites. These future studies could involve the use of newly available genomes (Liu et al. 2025; Alun et al. 2026) and the investigation of additional mechanisms, such as horizontal gene transfer (Liu et al. 2026) and transposable element methylation dynamics (Qiu et al. 2026). In particular, combining information on transposable elements, horizontal gene transfer, and transcription factor changes could provide valuable insights into the molecular mechanisms underlying social evolution.

Phylogenetic models indicate weak relationships between sociality and binding domains, as the social index modifying effect produced the best fitting model (Supplementary Table 1,Supplementary Table 2). We observed a positive shift in coefficients as species become more social. However, even with weakly regularising priors, multi-collinearity remains a problem for model interpretation. Larger orthogroups naturally contain more protein domains, and the number of DNA binding domains correlates with the number of activating domains as TFs possess both. Nevertheless, LOO model selection and ROPE results indicate that the social modifying effect is beneficial for model prediction, indicating socially correlated changes in TF domains. This is supported by the DOMRates results (Fig. 2B), which show that fusion events predominate in both termites and cockroaches (Fig. 2B). While major changes in domain arrangements occur during the social transition to termites (Mikhailova et al. 2024), this does not seem to be the case for TFs. This supports the findings from Simola et al. (2013) that a large change in regulation, in this case reductions rather than expansions, followed by lineage specific adaptations accompany social transitions.

When examining selection, similarities in TFs emerge between Hymenoptera and Blattodea, with relaxed selection being more prevalent than positive selection. Genes under relaxed and positive selection shared similar functions, playing crucial roles in neuronal development and memory formation such as *tango*, *klumpfuss*, *Lim3* and *CrebB*. Notably, the only positively selected orthogroup containing a DNA binding domain across all studied Blattodea is the neuro developmental zinc finger, *Lim3*. This consistent change in neuronal gene expression and cell number between reproductive castes is further supported by experimental evidence (Benjamin et al. 2024; Gospocic et al. 2021).

The global patterns found in Hymenoptera (Simola et al. 2013; Kapheim et al. 2015) of relaxed selection being more prominent than intensifying selection in social lineages held for Blattodea. This aligns with whole-genome studies showing relaxed selection in termites (Ewart et al. 2023; Roux et al. 2024) and inonotropic receptors (Mark et al. 2018). Interestingly, two genes under relaxed selection, *vri* and *snoo*, are involved in regulating the *dpp* pathway controling late stage wing development (Ohde et al. 2022). This fits the biology of neotenic (the process where juvenile traits are retained in adults) termite workers, which retain juvenile characteristics, while only alates have fully developed wings (Oguchi et al. 2021). Given the selection for wing development is primarily in the alates rather than workers, it makes sense that these genes are under relaxed selection.

In summary, while Hymenoptera shows an increase in transcription regulation with increased TF binding sites, Blattodea exhibit the opposite with fewer enriched sites (particularly in TF promoters) and fewer TFs, particularly those losing activating domains. However, both orders share a pattern of lineage-specific changes, with social species more likely to have more unique DNA activating domains within TF containing gene families (Fig. 3B). What also has to be considered, as stated earlier, is the difference in the number of species that can be investigated in Blattodea compared to Hymenoptera and the variation in the Blattodea genome qualities. While the quality of the genomes used is generally very good, variation in annotation quality, particularly in *M. natalensis *, could have influenced transcription factor identification and orthogroup-size estimates. As *M. natalensis *is the only higher termite in the dataset, any patterns associated with higher termites should be interpreted with caution, as they may also reflect species-specific or annotation-related effects. Future studies may have the computational capacity to look at if these are larger evolutionary patterns or lineage specific changes. By identifying a link between TF gene family diversity and sociality in termites. Future studies must look at these changes in regulatory mechanisms in combination, rather than in isolation, to better understand social transitions.

## Supplementary Information

Below is the link to the electronic supplementary material.

Supplementary file 1 (pdf 1646 KB)

## Data Availability

Scripts and data available at https://doi.org/10.6084/m9.figshare.c.4902573.
