# Supplementary material for: Transcription Factor Evolution Associated with Social Transitions in Blattodea
Source: J Mol Evol. 2026 Jul 23;94(4):655–67. doi: 10.1007/s00239-026-10328-1 (PMC13433471; doi:10.1007/s00239-026-10328-1)
Supplement: Supplementary file 1 [file 239_2026_10328_MOESM1_ESM.pdf]

Supplementary materials

Model	elpd_diff	se_diff	Stacking Weight
Unique domains model and social index modifier with activating domains	0.0	0.0	0.683
Unique full model	-6.6	5.5	0.227
Unique domains model and social index modifier with DNA binding domains	-11.5	5.2	0.000
Unique domains model and no modifier	-7.2	5.5	0.000
Unique only DNA binding domains	-895.5	68.8	0.066
Unique only activating domains	-2641.3	97.6	0.023

Table 1. Table showing the moment matched LOO comparison between phylogenetic models using the total number of unique DNA binding domains and total number of unique activating domains as predictors. Model comparison using ELPD differences, standard errors, and stacking model weights.

Model	elpd_diff	se_diff	Stacking Weight
Total no domains and modifier with social index	0.0	0.0	0.536
Total no activating domains responding with social index	-6.3	6.7	0.167
Total no binding domains domains responding with social index	-6.4	7.6	0.297
Total no domains and no modifier	-17.1	8.7	0.000

Table 2. Table showing the moment matched LOO comparison between phylogenetic models using the total number of activating and DNA binding domains as a predictor. Model comparison using ELPD differences, standard errors, and stacking model weights.

Table 3. Model Summary for `Total_full_model`

**Attribute**	**Details**
Family	Negative Binomial
Links	μ = log; shape = identity
Formula	Gene_counts ∼ bind_cof * Social_index + bind_within_spec<br>+ inter_cof * Social_index + inter_within_spec<br>+ (1 | gr(Phylo_index, cov = cov))<br>+ (1 | Species_index) + (1 | Ortho_groups)
Observations	17820
Chains	4
Iterations per chain	2000 (1000 warmup)
Total post-warmup draws	4000
**Multilevel Hyperparameters**	
Ortho_groups (n=1620)	sd(Intercept): 0.91 [0.87, 0.96], Rhat=1.00, ESS=1173/2079
Phylo_index (n=11)	sd(Intercept): 0.22 [0.01, 0.75], Rhat=1.00, ESS=1502/1206
Species_index (n=11)	sd(Intercept): 0.14 [0.00, 0.54], Rhat=1.00, ESS=1177/1597

Table 3 – continued from previous page

Attribute	Details
**Regression Coefficients**	
Intercept	-1.42 ± 0.47 [–2.31, –0.48], Rhat=1.00, ESS=2623/2655
bind_cof	0.29 ± 0.42 [–0.53, 1.11], Rhat=1.00, ESS=1763/2033
Social_index1	0.27 ± 0.45 [–0.62, 1.11], Rhat=1.00, ESS=3377/3112
Social_index2	–0.01 ± 0.48 [–0.94, 0.95], Rhat=1.00, ESS=5280/2479
Social_index3	0.05 ± 0.50 [–0.94, 1.04], Rhat=1.00, ESS=6280/2963
bind_within_spec	0.05 ± 0.00 [0.04, 0.05], Rhat=1.00, ESS=3089/3195
inter_cof	0.53 ± 0.44 [–0.37, 1.39], Rhat=1.00, ESS=4120/3228
inter_within_spec	0.13 ± 0.00 [0.12, 0.14], Rhat=1.00, ESS=3490/3601
bind_cof:Social_index1	–0.16 ± 0.40 [–0.94, 0.63], Rhat=1.00, ESS=1895/2045
bind_cof:Social_index2	–0.15 ± 1.50 [–3.42, 2.79], Rhat=1.00, ESS=1609/1703
bind_cof:Social_index3	0.05 ± 0.60 [–1.13, 1.13], Rhat=1.00, ESS=1474/2011
Social_index1:inter_cof	–0.09 ± 0.64 [–1.19, 1.16], Rhat=1.00, ESS=1721/1977
Social_index2:inter_cof	0.09 ± 4.65 [–9.03, 9.94], Rhat=1.00, ESS=1737/1627
Social_index3:inter_cof	–0.53 ± 1.62 [–3.53, 2.73], Rhat=1.00, ESS=1789/1752
**Further Distributional Parameters**	
shape	1.78 ± 0.05 [1.67, 1.89], Rhat=1.00, ESS=2995/3194

Table 4. Model Summary for `Unique_interacting_social_model`

Attribute	Details
Family	Negative Binomial
Links	μ = log; shape = identity
Formula	Gene_counts ∼ U_bind_cof + U_bind_within_spec + U_inter_cof * Social_index + U_inter_within_spec + (1 | gr(Phylo_index, cov = cov))

Table 4 – continued from previous page

**Attribute**	**Details**
	+ (1 | Species_index) + (1 | Ortho_groups)
Observations	17820
Chains	4
Iterations per chain	2000 (1000 warmup)
Total post-warmup draws	4000
**Multilevel Hyperparameters**	
Ortho_groups (n=1620)	sd(Intercept): 0.93 [0.89, 0.97], Rhat=1.00, ESS=800/1517
Phylo_index (n=11)	sd(Intercept): 0.19 [0.01, 0.49], Rhat=1.00, ESS=1305/1116
Species_index (n=11)	sd(Intercept): 0.09 [0.00, 0.28], Rhat=1.00, ESS=1004/1699
**Regression Coefficients**	
Intercept	-1.69 ± 0.26 [–2.18, –1.13], Rhat=1.00, ESS=3356/2816
U_bind_cof	0.77 ± 0.46 [–0.17, 1.66], Rhat=1.00, ESS=3719/2730
U_bind_within_spec	1.21 ± 0.02 [1.17, 1.24], Rhat=1.00, ESS=2513/3047
U_inter_cof	0.55 ± 0.46 [–0.34, 1.48], Rhat=1.00, ESS=4935/2471
Social_index1	0.39 ± 0.39 [–0.43, 1.10], Rhat=1.00, ESS=2592/2760
Social_index2	–0.15 ± 0.48 [–1.11, 0.82], Rhat=1.00, ESS=3140/2554
Social_index3	–0.25 ± 0.43 [–1.06, 0.64], Rhat=1.00, ESS=2605/2778

Table 4 – continued from previous page

Attribute	Details
U_inter_within_spec	0.84 ± 0.02 [0.80, 0.88], Rhat=1.00, ESS=1373/2130
U_inter_cof:Social_index1	–0.33 ± 1.23 [–2.58, 2.42], Rhat=1.00, ESS=2302/2355
U_inter_cof:Social_index2	0.76 ± 1.59 [–2.39, 3.87], Rhat=1.00, ESS=2631/2302
U_inter_cof:Social_index3	0.73 ± 1.46 [–2.18, 3.59], Rhat=1.00, ESS=2364/2416
**Further Distributional Parameters**	
shape	3.50 ± 0.13 [3.25, 3.76], Rhat=1.00, ESS=5142/3029

Table 5. Orthogroups inferred to be under relaxed selection, including Drosophila melanogaster best-hit annotations, selection parameters, and significance values.

Orthogroup	Dmel gene	Protein ID	K	P-value	FDR
OG0004628	vrille (vri)	FBpp0078706	0.5095	1.97e-07	4.44e-06
OG0004847	Snoo (Snoo oncogene)	FBpp0111629	0.5839	0.00246	0.0158
OG0005068	COX10	FBpp0079582	0.2181	2.71e-05	4.06e-04
OG0005261	klumpfuss (klu)	FBpp0303113	0.3999	0.0006336	0.00654
OG0005957	CEP104	FBpp0080789	0.6805	0.00238	0.0158
OG0006105	Vps16B	FBpp0084899	0.3576	5.79e-09	2.60e-07
OG0006401	tango (tgo)	FBpp0081483	0.6077	7.26e-04	0.00654

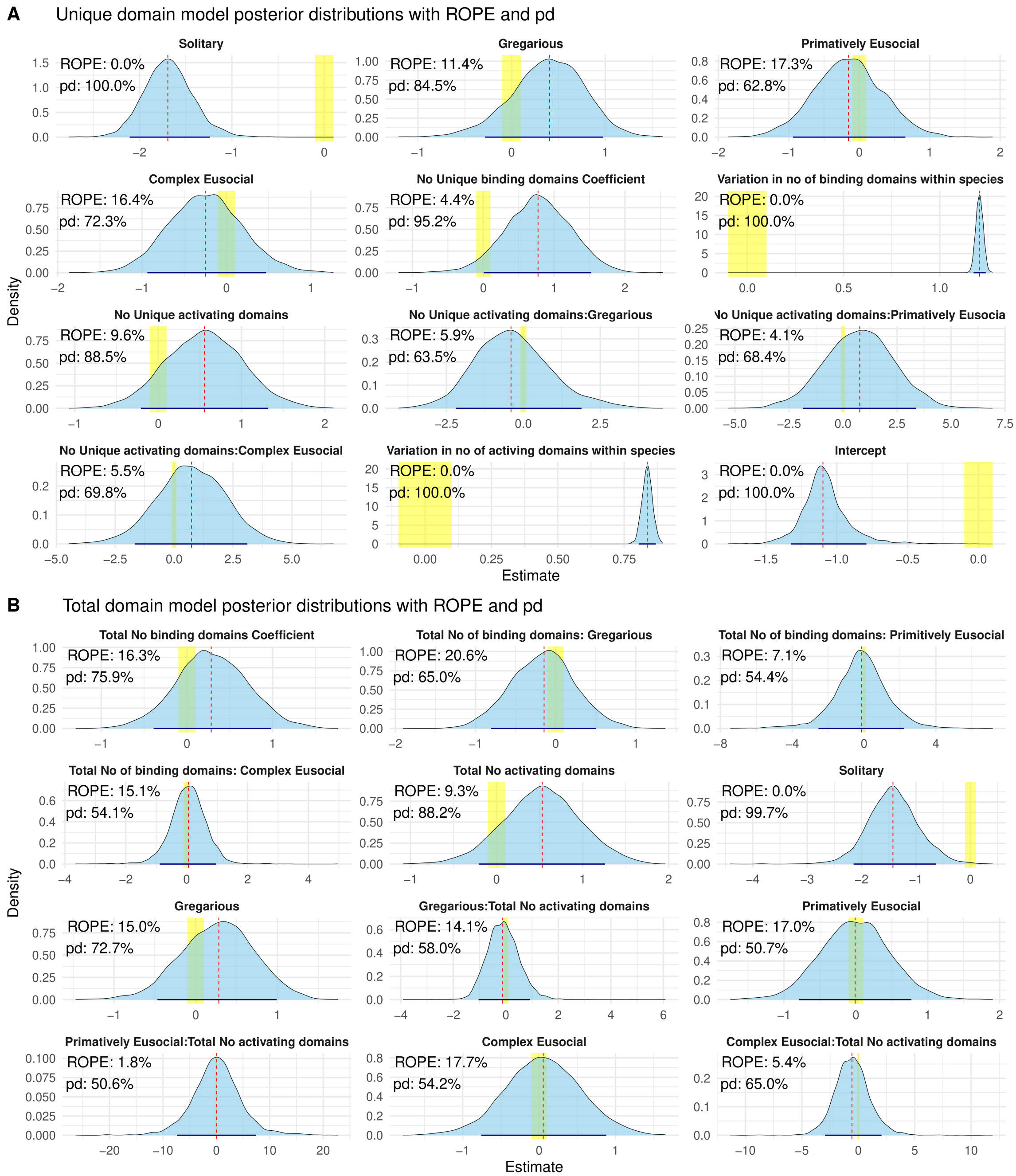

Figure 3. Here we are some of the results of the phylogenetic modelling looking at how total number of domains and the number of unique domain predicts the gene size of an orthogroup, which can be seen in Table 4 and 4. We are showing the posterior distributions of a selected number of coefficients of the best fitting models, based upon LOO comparisons. The yellow region is the region of posterior equivalence (ROPE) between -0.05 and 0.05 with the percentage being the percentage of the posterior distribution that lies within this region. pd percentage indicates the probability of direction (PD). A) Shows the best fitting model based upon the total unique number of domains within the gene family. B) Shows the best-fitting model based upon the number of unique domains within the Gene family. There is a positive shift from interaction between the No Unique activating domains and Gregarious species and Eusocial species. Indicating there are more unique activating domains in Eusocial species orthogroups.

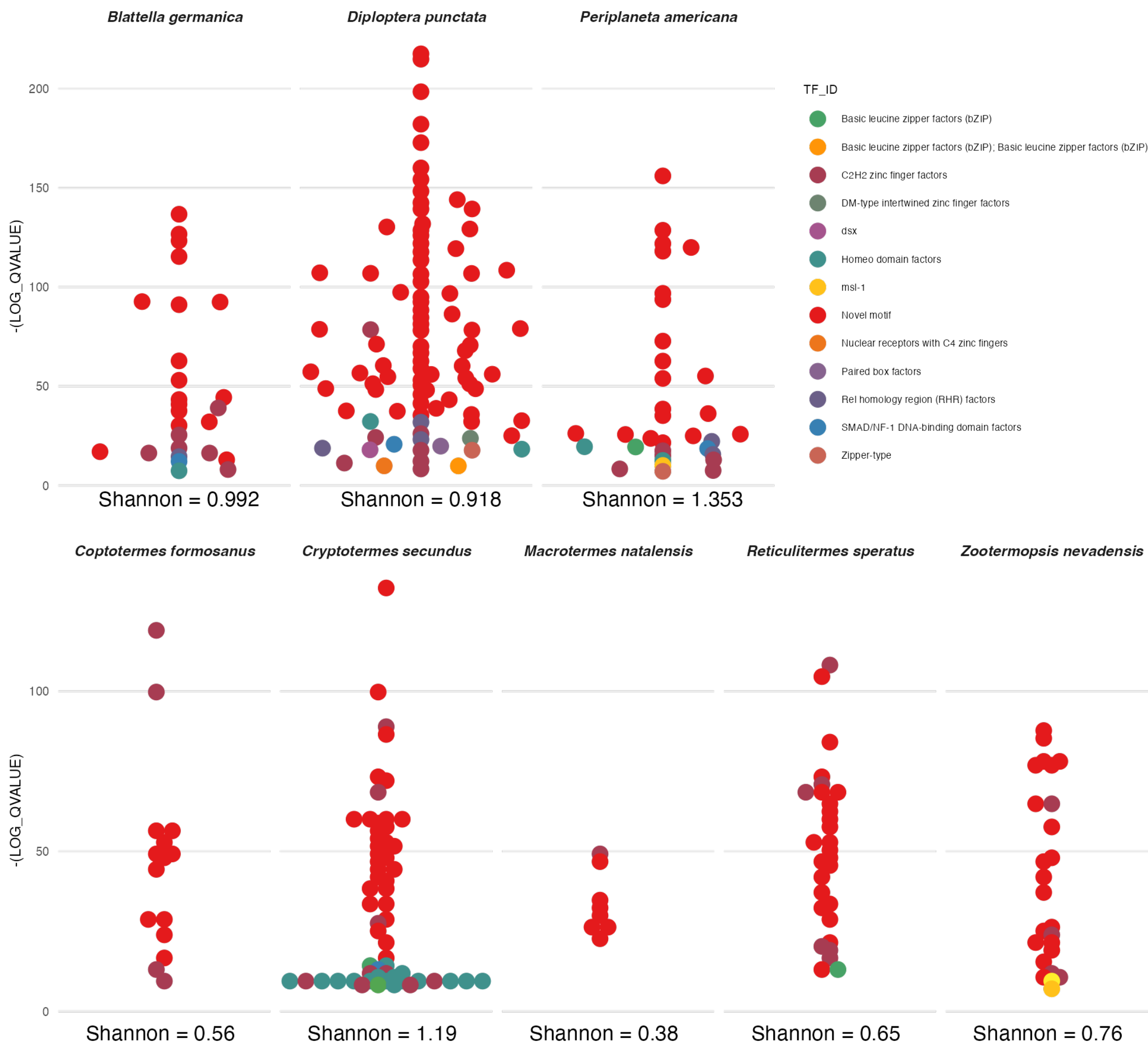

Figure 4. Enrichment of DNA binding domain motifs in 2kbp promoter regions of TFs in cockroaches as well as termites, identified using SEA from the XStreme package. The Shannon diversity index is displayed for each species. The y axis is the - log q value showing the significance of motif enrichment.
